# Anaesthetic Approach to Enhanced Recovery after Surgery for Kidney Transplantation: A Narrative Review

**DOI:** 10.3390/jcm11123435

**Published:** 2022-06-15

**Authors:** Slawomir Jaszczuk, Shweta Natarajan, Vassilios Papalois

**Affiliations:** 1 Department of Anaesthesia, Imperial College, London W12 0HS, UK; shweta.natarajan2@nhs.net; 2 Department of Surgery, Imperial College, London W12 0HS, UK; vassilios.papalois@nhs.net

**Keywords:** kidney, transplantation, enhanced recovery after surgery, fast track, green anaesthesia

## Abstract

Enhanced recovery after surgery (ERAS) protocols are designed to reduce medical complications, the length of hospital stays (LoS), and healthcare costs. ERAS is considered safe and effective for kidney transplant (KTx) surgery. KTx recipients are often frail with multiple comorbidities. As these patients follow an extensive diagnostic pathway preoperatively, the ERAS protocol can ideally be implemented at this stage. Small singular changes in a long perioperative pathway can result in significant positive outcomes. We have investigated the current evidence for an ERAS pathway related to anaesthetic considerations in renal transplant surgery for adult recipients.

## 1. Introduction

Enhanced recovery after surgery (ERAS) is a protocol of components related to preoperative, intraoperative and postoperative care. The objective of the protocol is to improve patient recovery, reduce the risk of complications and readmissions, facilitate earlier discharge, and reduce healthcare cost. This may also be one step towards a sustainable healthcare practice.

The pioneer of ERAS is Danish professor Henrik Kehlet, who published several articles on fast-track protocols in colorectal surgery in 1990s. The concept of fast-track surgery combines different strategies based on evidence-based medicine. The integration of current knowledge and its application to clinical practice lead to minimization both of the physiological stress response associated with surgery and of the potential risk of organ dysfunction, thereby increasing long-term survival [1]. The effectiveness of these programs is highly dependent on a hospital’s ability to implement certain guidelines [2].

The ERAS Society consensus guidelines have been developed in multiple specialties, but there is no review on ERAS programs for kidney transplant recipients. Some authors have reported on the development of their own ERAS pathway [3], and on the successful implementation of the ERAS program in kidney transplants (KTx) that show a significant reduction in the length of hospital stays (LoS) and costs, with no increase in the frequency of complications [4,5,6].

The development and implementation of ERAS protocols requires coordinated input from a multidisciplinary team. We will explore the available evidence as to how an anaesthetic appraisal and intervention can contribute to the development of robust protocols in KTx.

Our review aims to highlight an anaesthetic approach for an ERAS pathway for KTx recipients through exploring recent available evidence on preoperative, intraoperative, and postoperative considerations. This narrative review can be incorporated and developed into a robust ERAS protocol for KTx. This narrative review will improve safety and efficiency through streamlining patients’ recovery using pre-habilitation, optimal intraoperative management, and multi-disciplinary post-surgical care, as well as reducing patient LoS and overall cost.

Although the ERAS protocol provides relevant recommendations for preoperative optimisation, it is important to refer to this protocol as a contributing factor when assessing the readiness of the patient for surgery. The final decision on the inclusion, retention, or removal a patient from the transplant waiting list is a multicriteria decision and the clinical needs of the patient must be considered.

## 2. Preoperative Care

### 2.1. Medical Optimisation

Maintaining patient readiness for transplantation is challenging [7,8]. Assessment and optimisation of cardiac, respiratory, and other conditions decrease the likelihood of adverse events [9,10]. This includes the implementation of high-quality evidence-based medical guidelines, demonstrating a reduction in morbidity and mortality [9,10,11,12]. The anaesthetist can play an important role in optimising outcomes as part of a team.

#### 2.1.1. Cardiovascular Disease

The risk of cardiovascular disease is 5 to 30 times higher in the ESRF population compared to the general population [13]. Other comorbidities typical for chronic kidney disease (CKD) patients are HTN (90% patients), ischaemic heart disease (IHD) (25% patients), diabetes mellitus (DM) (30% patients), and obesity (30% patients). Patients with symptoms of heart failure, valvular disease, angina, and arrythmia should be assessed by a cardiologist [14,15,16].

##### Electrocardiogram

Given the high cardiovascular risk, evaluation of the electrocardiogram (ECG) and cardiac history is essential. Preoperative ECG changes are associated with increased post-transplant cardiovascular risk [15].

##### Echocardiogram

Echocardiography is recommended for patients with ventricular impairment or valvular disease, and patients at risk of pulmonary hypertension [15,16]. Moderately stenotic valvular lesions require an annual echocardiographic assessment [15]. Patients with severe valvular lesions should be assessed by a cardiologist and managed according to local guidelines [16].

##### Non-Invasive Coronary Tests

Non-invasive coronary stress tests may be considered in potential KTx recipients who have multiple risk factors for coronary artery disease, such as DM, HTN, left ventricle hypertrophy, age greater than 60, dyslipidaemia, and smoking. However, it is not clear which test should be used for coronary artery assessment in asymptomatic patients, or whether repeat testing for myocardial ischaemia provides any benefit to patients on a waiting list [7,15,16,17]. There is poor correlation between non-invasive tests and coronary angiography with clinical cardiac events [16,17]. Even if significant coronary stenosis is identified, there is a lack of evidence for the benefits of revascularization [14,15,17].

##### Cardiac Troponins

Evaluation of cardiac troponins (cTns) can be considered for risk assessment, as persistently elevated cTns in ESRF patients correlate with increased mortality [7,15].

##### Percutaneous Coronary Intervention

It is recommended that patients prepared for percutaneous coronary intervention (PCI) who are likely to undergo KTx surgery in the next 12 months be considered for balloon angioplasty or bare metal stent (BMS), rather than a routine drug-eluting stent (DES), followed by 4 to 12 weeks of dual antiplatelet therapy (DAPT). Patients with DES should have thienopyridines stopped before surgery and aspirin continued. It is reasonable to restart thienopyridines as soon as possible. Transplantation within four weeks after coronary revascularisation with balloon angioplasty, three months after BMS placement, and twelve months after DES placement is not recommended [15]. Beta-blockers should be continued in the preoperative and postoperative period. Initiating beta-blocker therapy may benefit patients at increased cardiovascular risk [16,17].

##### Screening

Since patients could be on the waiting list for some years prior to KTx, repeated cardiac evaluation is recommended, especially in patients with unstable coronary syndromes, decompensated heart failure, significant valvular disease, and arrhythmias. The optimal frequency of screening asymptomatic patients for myocardial ischaemia is unknown and not supported by any evidence [7,15,16,17]. The standard recommendation is to repeat cardiac stress testing every year, especially in patients with diabetes. [8].

##### Pulmonary Hypertension

ESRF patients have a two to eight times higher risk of developing pulmonary hypertension (PHT) due to multiple risk factors, including arteriovenous (AV) fistulas used for dialysis, high cardiac output state, fluid overload, anaemia, left ventricle (LV) impairment, and endothelial dysfunction. Nocturnal hypoxaemia, often diagnosed in this group of patients, also plays an important role. Patients with pulmonary systolic pressure (PSP) higher than 35 mmHg have significantly greater mortality than patients with PSP below 35 mmHg (30.8% vs. 3.5%) [18]. The severity of PHT is related to the flow and duration of the AV fistula. Large flows or high cardiac output may require reduction or closure of the fistula. Recipients with significant pulmonary hypertension may require vasodilator therapy. Pulmonary artery pressures reduce significantly one year after KTx. Living donor grafts are preferable in KTx recipients with PHT to minimise risk of early graft dysfunction [18,19]. Recipients with PSP greater than 45 mmHg should be assessed by a cardiologist [16].

##### Infective Endocarditis

The risk of developing infective endocarditis is 30–100 times higher in patients on haemodialysis (HD), with a 1-year mortality of 40–60%. Synthetic catheters and AV fistula grafts are risk factors and should be removed when valvular disease is diagnosed [18].

#### 2.1.2. Pulmonary Disease

Pulmonary function testing is recommended in all KTx recipients with impaired functional capacity, respiratory symptoms, or known pulmonary disease [16].

The literature reviewed in this article focused primarily on heart and lung problems, considering these to be the leading medical causes of death in KTx recipients. A detailed description of other medical conditions is a broader topic that is beyond of the scope of this review.

#### 2.1.3. Reasons to Delay Transplant

In certain circumstances, deferment of surgery should be considered to allow for better optimisation. Delaying transplantation is recommended in patients with substance abuse issues that put a candidate at unacceptable risk, active symptomatic cardiac disease not evaluated by a cardiologist, recent stroke, acute symptomatic inflammatory gastrointestinal disease, or active peripheral arterial disease [16]. However, before removing a patient from the waiting list, other factors must be taken into account, such as the urgency for the transplant.

There is lack of evidence on timing between COVID-19 infection and KTx [20]. Guidelines for the general population suggest avoiding surgery within seven weeks of infection [21]. However, KTx is not an elective surgery, and the decision must be weighed against the clinical needs for the surgery.

We suggest repeat cardiac evaluation, especially in patients with unstable coronary syndromes, decompensated heart failure, significant valvular disease, and arrhythmias. Echocardiography is recommended for patients with ventricular impairment or valvular disease, and patients at risk of pulmonary hypertension. Recipients with PSP greater than 45 mmHg should be assessed by a cardiologist. Pulmonary function testing is recommended for symptomatic patients.

Key articles: [15,16,17].

### 2.2. Patient Education

Preoperative education is a fundamental part of the anaesthetic pre-evaluation, since it allows informed consent and preparation, and addresses physical, psychological, and social needs. This is especially relevant in managing patients with depression or unrealistic expectations. Patient education may have a positive effect on preoperative anxiety [11]. Quitting smoking and risky drinking is beneficial, and these benefits are supported by evidence.

Smoking is a risk factor for pulmonary complications. Smokers in the general population have slightly increased mortality at three days post-surgery, greater airway irritability, and higher incidence of cough, laryngospasm, and delayed healing of wounds [22]. The optimal time to quit smoking is unknown. Kidney Disease: Improving Global Outcomes (KDIGO) recommends smoking cessation for at least four weeks prior to surgery. The risks decrease with each additional week of smoking cessation [16,23]. However, another trial of kidney transplant patients suggests longer periods for smoking cessation [24]. Kidney allograft recipients who continued to smoke have a higher rate of post-transplant cancer and cardiac events, increased incidence of rejection at one year, and higher risk of death and overall graft failure [25,26,27].

All KTx recipients should be screened for tobacco use, and users offered a program for tobacco cessation [14,16]. Nicotine replacement therapy is effective and should be offered to any nicotine-dependent patient. Some authors recommend the use of e-cigarettes as an aid in quitting smoking. There is not enough evidence to support using e-cigarettes after solid organ transplantation [24,25]. Smokers with a history of more than 30 pack years should have computed tomography as a cancer screening test [16].

Risky alcohol consumption is defined as alcohol intake equivalent to more than 3 alcoholic units (24 g) per day or 21 units per week. One unit of alcohol consists of 12 g of ethanol. Risky alcohol drinking habits have a detrimental effect on patients’ health. The Cochrane Database Systematic Review revealed that postoperative complication rates in surgical patients increased by approximately 50% with an intake of more than two to three units a day [28]. The complication rate increases by 300% if consumption is more than five units per day; this includes adverse cardiovascular events, bleeding, and infection. [28]. Alcohol-dependent KTx recipients have a higher risk of graft failure and mortality than patients who are not alcohol-dependent [29]. However, one study showed that moderate alcohol consumption (10–30 g/day) had a protective effect on allograft recipients. Moderate alcohol intake is associated with a lower risk of post-transplant diabetes mellitus (PTDM) and all-cause mortality compared with abstainers [30].

Frailty represents vulnerability to stressors and deterioration of physical reserves. Frailty is very common in ESRF patients and is a predictor of adverse outcomes such as delayed graft function (DGF), postoperative delirium (POD), LoS, early readmission rate, and death [31,32]. Among KTx recipients, 7% have cognitive impairment and 15% are frail [33,34], with frail patients experiencing cognitive decline after transplantation and not recovering to baseline levels at one and four years. Although there may not be sufficient evidence, there are multiple measures that can be carried out in frail patients to optimize the implementation of the ERAS protocol [35,36,37].

We suggest that patients should quit smoking at least four weeks before surgery and smokers should be offered nicotine replacement therapy. Risky drinking should cease for four to eight weeks before surgery. Preoperative frailty screening is recommended for risk assessment.

Key articles: [14,16,29].

### 2.3. Pre-Habilitation

Pre-habilitation is a program that improves the patient’s functional capacity, allowing for improved responses to perioperative stress. Modern pre-habilitation aims not only for physical fitness, but also for good nutrition, stress management, and promotion of healthy habits. Patients should understand the benefits of this preoperative approach [38].

Physical fitness is reduced in CKD patients. The main causes of physical deterioration are anaemia, loss of muscle mass [39], and post-dialysis fatigue syndrome [40]. Insulin resistance, acidosis, and enhanced muscle proteolysis are typical for patients with ESRF. Exercise is positively correlated with tissue sensitivity to insulin [39]. Regular High Intensity Training (HITT) has beneficial effects on adults with CKD with regard to physical condition, walking capacity, cardiovascular dimensions, health-related quality of life, and nutritional parameters. Positive effects can be observed after three months of regular exercise [39]. Pre-habilitation lowered LoS among KTx recipients [41]. Exercise therapy is recommended for HD patients as it can improve exercise tolerance and walking ability [40]. Patients should exercise two to three times a week at first, increasing frequency and intensity over time. The types of exercise should include aerobic, resistance, and flexibility. [40,42]. There is insufficient evidence to recommend exercise for people on peritoneal dialysis due to a lack of studies [40].

We suggest that KTX recipients should be offered exercise therapy lasting more than 30 min two to three times a week.

Key articles: [39,40].

### 2.4. Improving Nutritional Status

According to the definition of the European Society for Clinical Nutrition and Metabolism (ESPEN), malnutrition is defined as body mass index less than 18.5 kg/m^2^, or, combined: weight loss > 10%, or >5% over 3 months and reduced BMI or a fat-free mass index (FFMI—the amount of fat-free mass in relation to height and weight). It is a common problem in patients with CKD [43,44]. The inflammatory process, loss of appetite, uraemia, fasting before procedures, and the presence of comorbidities contribute to a poor nutritional state. More than half of hospitalized patients requiring HD are malnourished. HD removes four to nine grams of amino acids during each session [45]. The main nutritional interventions in uraemic patient are to minimise uraemic toxicity and avoid malnutrition [46].

After general surgery, malnourished patients have higher postoperative mortality, morbidity, risk of infection, and LoS, and higher hospital costs. Their readmission rate within 30 days is doubled [47]. Malnourished patients with renal disease have poor outcomes and higher mortality than non-malnourished uraemic patients. Preoperative hypoalbuminemia is one of the risk factors. Serum albumin concentration < 30 g/L is considered the strongest predictor of death from malnutrition in HD patients. There is no single method to stratify risk in chronically malnourished uraemic patients [46].

Patients with CKD tend to have a lower energy intake than prescribed. Protein intake is usually higher than recommended in uraemic individuals, and lower than recommended in the dialysis group [46]. The UK Renal Association recommends an energy intake of 30 to 40 kcal/kg ideal body weight (IBW)/day and a protein intake of 0.8 to 1.0 g/kg IBW/day for patients with CKD in stages 4 to 5, who are not on HD, 1.1 to 1.4 g/kg IBW/day for patients on HD, and 1.0 to 1.2 g/kg IBW/day for patients on peritoneal dialysis (PD) [47,48].

Patients should be evaluated before interventions, and those with nutritional risk before major surgery should receive oral nutritional supplements or other forms of feeding (enteral or parenteral) for at least seven days [47].

Protein-based oral nutrition is often offered to CKD patients who cannot meet their dietary needs. This supplement increases the levels of serum albumin and prealbumin, and the circumference of the middle arm. It is not clear whether such an approach improves overall nutritional status or has relevant clinical outcomes, such as death [49]

Given the relationship between an increased risk of adverse outcomes and malnutrition, these malnourished patients with renal insufficiency should be referred to a dietician for optimisation [45].

Obesity, defined as a body mass index (BMI) ≥ 30 kg/m^2^, is associated with an increased risk of cardiovascular complications and mortality in KTX recipients. Weight and waist circumference should be measured for all patients. Obese allograft recipients should be offered a diet and behaviour-modifying lifestyle. There is insufficient evidence to support pharmacological or surgical treatment of obesity [14,16].

We suggest that kidney transplant recipients should be evaluated, and malnourished patients should be referred to a dietician. Diet and exercise should be offered to all obese KTx recipients.

Key articles: [16,45,46,47].

### 2.5. Carbohydrate Drink before Surgery

Currently, there is a lack of data on the impact of carbohydrate drinks on kidney transplant recipients. Recommendations from other types of major surgery can be extrapolated.

A preoperative drink containing at least 45 g of carbohydrate is recommended in patients undergoing major surgery, except in patients with severe insulin-dependent DM and anticipated delayed gastric emptying [43,44]. Fruit-based lemonade may be considered as an alternative. The use of homemade products like sweetened tea has not been studied [43].

Preoperative carbohydrate drinks have proven to reduce postoperative nausea and vomiting (PONV), shorten the length of stay, and improve insulin resistance by increasing sensitivity by 50% [43,44,50]. Additionally, they shift cellular metabolism to an anabolic state that allows for better protein retention and preservation of lean body mass. This treatment reduces the prevalence of preoperative thirst, hunger, and anxiety. Preoperative intake of 400 mL of carbohydrate drink before surgery or 800 mL the night before does not increase the risk of aspiration [43].

Patients who cannot be enterally fed can receive parenteral loading. The dose of 1.5–2 g/kg of glucose and 1 g/kg of amino acids has a positive impact on adaptation to perioperative stress [43].

Unnecessary prolonged preoperative fasting does not reduce the rate of complications and should be avoided. In patients with minimal risk of aspiration, unrestricted access to solids and clear fluids should be allowed up to 6 h and 2 h, respectively, before anaesthesia [43,44].

We suggest that a drink containing at least 45 g of carbohydrates should be offered to all patients, except those with diabetes mellitus and anticipated delayed gastric emptying.

Key articles: [43].

### 2.6. Preoperative Anaemia Correction

Most patients with CKD have anaemia, with the prevalence being twice that of the general population, and six times higher in stage 5 CKD [51]. Adverse outcomes caused by anaemia and blood transfusion are well known. Even a small amount of transfused blood in the general population increases mortality, surgical site infection, pneumonia, and sepsis [52]. Blood transfusion can cause allosensitisation, iron overload, and transmission of viral disease, and should be avoided if possible in potential KTx recipients [53]. Anaemia often occurs in HD patients in whom blood sampling and clotting of dialysis circuits cause significant blood loss [51]. Anaemia in patients with CKD should be managed if the haemoglobin (Hb) level falls to 110 g/L, or when patients become symptomatic [54].

Responsiveness to iron treatment should be assessed, and deficiency evaluated [52,54]. People not receiving HD should be offered oral iron supplementation or intravenous (IV) iron if the oral form is not tolerated. Patients receiving HD should be offered IV iron first, unless contraindicated [54]. IV iron is more effective than oral iron in anaemia treatment and has reduced side effects such as constipation, diarrhea, nausea, and vomiting. There is insufficient evidence regarding the superiority of parenteral iron compared to oral iron for quality of life, death rate, or death from heart disease [55]. Patients receiving IV iron supplementation should have iron levels checked no sooner than one week after treatment. Routine monitoring should be performed every one to three months [54].

Erythropoietin (EPO) deficiency is one of the factors that causes anaemia in patients with CKD. EPO treatment can prevent blood transfusion and its complications, which is crucial for KTx recipients. EPO therapy improves quality of life and cognitive function, and helps regression of left ventricular hypertrophy; however, no mortality benefit has been demonstrated. EPO can cause various complications such as hypertension, seizures, thrombosis of arteriovenous fistula, and increased risk of stroke [53,56]. It is recommended to initiate treatment with EPO in CKD patients who will benefit in terms of quality of life and physical function [53,54,57], and whose haemoglobin levels are between 90 and 100 g/L [56]. Patient preferences, routes of administration, EPO availability and cost [54] should be considered. When using erythropoietin stimulating agents (ESA), Hb corrections to normal values are not recommended, as complete correction of anaemia increases mortality. The aim should be to increase the Hb concentration by 10 to 20 g/L/month [54].

Vitamin C, folic acid, carnitine, and androgens should not be used to treat anaemia in CKD patients. Treatment of hyperparathyroidism improves the management of anaemia [53,54].

Anaemic patients have reduced graft survival and increased mortality after KTx. Anaemia treatment should commence after transplantation with a target level of 12.5–13 g/dL [58].

We suggest avoiding blood transfusion in KTx recipients. Response to iron treatment should be assessed in anaemic patients. ESA treatment should be considered for anaemic KTx recipients with Hb levels 90–100 g/L; the benefits of reducing blood transfusion should be balanced with the risk of side effects. ESA should not be started in patients with iron deficiency.

Key articles: [53,54,57].

### 2.7. Anxiolysis

Anxiety begins long before surgery and peaks just prior to intervention. Anxiety can complicate anaesthetic induction, delay postoperative recovery, and increase the incidence of postoperative nausea and vomiting [59]. The level of anxiety correlates with postoperative pain scales in patients after craniotomy [60]. However, patients who are excessively sedated prior to anaesthesia may require increased care in the postoperative period. The relationship between anaesthetic premedication and graft function has been poorly evaluated in the literature [59].

Monitoring anxiety symptoms is important for tailored anxiolysis. The State Trait Anxiety Inventory (STAI) questionnaire is one of the available scales that allows the level of anxiety to be measured [60].

Preoperative anxiety can be reduced by providing comprehensive information and psychological support [59] There are currently no data on an effective premedication regime in KTx recipients.

Midazolam is metabolised in the liver to its active form α-hydroxymidazolam. Midazolam protein biding and elimination of α-hydroxymidazolam are reduced in CKD and cause prolonged sedation [61,62]. Benzodiazepines should be used with caution, especially in elderly patients, as they are at high risk of cognitive decline and falls [63,64]. This situation encourages the search for other premedication options.

Dexmedetomidine is well known for its anxiolytic, sedative, analgesic, anaesthetic-sparing, and respiratory-sparing effects, and can be used as an alternative to midazolam. Perioperative dexmedetomidine use inhibits the stress response, limits inflammation, and protects immune function [65,66,67,68,69]. However, dexmedetomidine in elderly patients causes hypotension [70]. Dexmedetomidine can be used in patients with ESRF [71,72].

Melatonin production and cycling are impaired in ESRF patients [73]. Compared to a placebo, Melatonin, when given as pre-medication in surgical patients, can reduce pre- and postoperative anxiety and pain, and improves recovery [74,75]. The antioxidant properties of melatonin could protect against cyclosporin nephrotoxicity [76], reduce ischemic-reperfusion injury [77] and graft rejection [76,78]. These properties appear at doses much higher than those used for sedation in patients after transplantation [76,77,78].

We suggest using anxiolytics for anxious patients before anaesthesia. The routine use of sedative agents should be avoided.

Key articles: [64,70,74].

## 3. Intraoperative Care

### 3.1. Standard Anaesthetic Protocol

ESRF patients suffer from many comorbidities, including DM and gastroparesis. Proton pump inhibitors (PPIs) or H2 inhibitors should be administered to patients with delayed stomach emptying to minimise aspiration risk [79].

HD is important as it optimises fluid volume, and normalises electrolyte imbalances and metabolic disorders. However, dialysis is not recommended on the day of surgery unless there is a high potassium level or fluid overload [79,80,81].

The venous cannula should not be inserted on the same side as the patient’s arterio-venous fistula [82]. The use of a central venous catheter is recommended, as a large proportion of patients have cardiac comorbidities; recipients can become unstable during surgery and require cardiovascular support [79]. General anaesthesia is routine and the most common technique for KTx [83].

#### 3.1.1. Induction of Anaesthesia

Propofol and thiopental are safe agents for induction. Ketamine should be avoided in IHD patients as the sympathetic stimulation can cause tachycardia [79,84]. Etomidate could be a safe choice for patients with IHD or impaired ventricular function due to its cardiovascular stability during induction. Etomidate can suppress the adrenal glands, but the use of perioperative methylprednisolone ensures an adequate steroid level [85].

#### 3.1.2. Opioids

Fentanyl and its analogues, such as alfentail, sulfentanil, and remifentanil, are safe options. Morphine should be avoided as it is metabolised to morphino-6-glucuronide, the concentration of which may increase in the ESRF [84].

#### 3.1.3. Maintenance of Anaesthesia

Compound A, created in the reaction of sevoflurane, has a nephrotoxic effect in rat studies. This has never been observed in humans with CKD and should therefore not influence the choice of inhalational anaesthetic [81]. Inorganic fluorides were responsible for renal failure following methoxyflurane anaesthesia. It has been suggested that sevoflurane may cause renal impairment, but the majority of other studies did not confirm sevoflurane nephrotoxicity [86,87]. Desflurane is considered a safe agent. The metabolism of isoflurane is very low and is unlikely to cause kidney damage. Propofol is metabolized in the liver and can be safely used for the maintenance of anaesthesia in patients with renal impairment [83,88,89].

#### 3.1.4. Neuromuscular Blocking Agents

Suxamethonium can be used for rapid sequence induction if a delay in stomach emptying is suspected. Suxamethonium can cause an elevated plasma potassium concentration. However, ESRF patients who have chronic hyperkalemia can tolerate a transient increase in potassium [81]. Rocuronium can be considered an agent for rapid sequence induction, but it shows prolonged action in patients with ESRF. The duration of action of vecuronium is also prolonged. Atracurium and cis-atracurium are inactivated through several pathways including Hoffmann elimination, and can be used in ESRF. However, ESRF causes low pH, which can alter the metabolism of atracurium and cis-atracurium. Laudanozine is an atracurium metabolite that causes seizures; it may accumulate in patients with ESRF. However, toxic levels of laudanozine have never been observed in humans. Cis-atracurium is more potent than atracurium, so less cis-atracurium is used and less laudanozine is produced during its metabolism. Mivacurium metabolism may be prolonged due to decreased plasma cholinesterase levels in dialysed patients. Pancuronium should be avoided due to its long-acting properties. Neuromuscular monitoring is recommended in all KTx patients [79,84,88].

Sugammadex is an effective agent which forms complexes with rocuronium and vecuronium to reverse neuromuscular blockades. These complexes are eliminated only by the kidneys and are retained in the body for a longer time in patients with renal impairment. The rocuronium–sugammadex complex can be eliminated only with certain forms of HD [87]. Dissociation of rocuronium causing a recurrence of the neuromuscular blockade has not been observed in current studies. Sugammadex appears to be safe and effective in kidney transplant recipients [90,91,92]. A recent meta-analysis suggests that recovery to train-of-four may be prolonged in patients with ESRF, and more data are needed to support the use of sugammadex in this group [92]. The routine use of quantitative neuromuscular monitoring is strongly recommended when sugammadex is used in patients with ESRD [90]. Neostigmine has a longer duration of action in ESRF and can cause bradycardia or heart block when combined with atropine, which has a shorter half-life [87]. Elimination of glycopyrronium is also impaired in CKD, which can be an advantage when used in combination with neostigmine.

#### 3.1.5. Diuretics

Mannitol is used routinely to improve kidney function after transplantation from a deceased donor, but there is great variation between centers [93]. Mannitol is an osmotic diuretic and oxygen radical scavenger. It causes vasodilation and increases tubular flow, preventing cast obstruction [80,94]. Administration of 250 mL 20% mannitol before reperfusion improves renal function and decreases the rate of DGF [84]. Mannitol improves renal blood flow, removes free radicals, and protects from post-transplantation tubular necrosis [79]. Excessive administration can be harmful, and can cause heart failure, pulmonary oedema, and hypertonic kidney failure [94]. Although studies showing the benefits of mannitol use are based on little evidence, the potential benefits outweigh the risks [94].

Furosemide (3–5 mg/kg) is administered routinely 10–15 min prior to clamp release. Nephroprotective effects may relate to counteractions of the antidiuretic hormone response and decreased renal oxygen consumption by blocking active tubular transport (which should provide resistance to ischaemia). However, studies have not shown strong evidence for the beneficial effects of furosemide in patients with acute kidney injury (AKI) [79,80,84]. Urinary responses to the bolus of 100 mg furosemide after renal vessels anastomosis can be used as risk stratification for DGF. Low urine output predicts which patients may require renal replacement therapy after surgery [95,96].

#### 3.1.6. Vasopressors

The transplanted organ has altered autoregulation. Organ function is dependent more on blood flow than blood pressure. HTN caused by high vascular resistance can potentially decrease blood flow and oxygen supply, which are essential for proper mitochondrial function [84]. Small doses of vasopressors can be used, as the risk of low blood pressure is greater than that of potential renal vasoconstriction [85]. There is a lack of evidence on vasopressor use and which to use as a first-line treatment [79]. Changes caused by graft storage and extra-corporeal preservation make the allograft unresponsive to dopamine. The empirical use of dopamine can be harmful and is not recommended. Noradrenaline, with its beta-adrenergic activity, seems to be a better option [80,81].

#### 3.1.7. Blood Pressure during Reperfusion

Intraoperative mean arterial pressure (MAP) lower than 70 mmHg during KTx appears to often be associated with DGF. Ideal MAP varies widely, from 80 mmHg to 110 mmHg, in clinical trials. Low MAP during reperfusion predisposes the patient to worsened kidney function. In the case of donor and recipient MAP mismatch, the function of the transplanted graft could be also impaired [79,97].

#### 3.1.8. Sodium Bicarbonate

Patients with CKD suffer from 2,3-diphosphoglycerate depletion, which causes an alteration in the haemoglobin–oxygen dissociation curve and reduces oxygen supply to tissues by Hb. The effect of a higher affinity of Hb for oxygen can be exaggerated by the administration of sodium bicarbonate, which should be used with caution. The base deficit should not be used as the sole indication for the use of sodium bicarbonate. A prolonged duration of cold graft ischaemia causes acute tubular necrosis with hyperkalaemia, oliguria, and metabolic acidosis. This scenario should be managed by dialysis, not sodium bicarbonate [85].

We suggest administering PPIs or H2 inhibitors for patients with gastroparesis. Anaesthetic agents that accumulate should be avoided in ESRF. Hypotension during surgery should be avoided, and MAP targets above 80 mmHg should be aimed for. Noradrenaline should be considered as a vasoconstrictor. Before reperfusion, 250 mL 20% mannitol should be transfused. Oliguric patients with post-transplant metabolic acidosis should be offered dialysis.

Key articles: [79,80,81].

### 3.2. Prevention of Postoperative Nausea and Vomiting

Prevention of PONV is an important element of the ERAS pathway. PONV delays discharge from recovery, worsens patient experience, increases hospital costs, and delays early oral intake, which is essential in all ERAS pathways.

KTx recipients receive methylprednisolone as an immunosuppressant. Methylprednisolone 40 mg IV is effective in preventing PONV, with side effects similar to that of dexamethasone [98]. Data on the prevention of PONV in KTx recipients are limited, and further research is required. There is no reason not to recommend the standard approach used for patients undergoing general surgery [98,99,100,101,102].

We suggest risk stratification and PONV prophylaxis for all patients.

Key articles: [98,99,100].

### 3.3. Perioperative Fluid Management

Fluid therapy during KTx is among the most challenging clinical tasks. Optimal fluid management has been shown to decrease DGF after KTx, which is associated with improved patient survival and long-term function, and decreased acute rejection [103]. The choice between the use of vasopressors and fluids for hypotension is too simplistic in most cases. Implementing cardiac output monitoring could help guide adequate fluid resuscitation [104,105]. The general approach to fluid resuscitation in patients undergoing major surgery based on current evidence should be: “as much as required, as little as possible” [105].

HD patients are more likely to have microinflammation, and glycocalyx and endothelial dysfunction. This makes them more susceptible to extracellular volume and salt overload [105]. A randomised study, using a porcine renal transplant model, compared individualized goal-directed fluid therapy (IGDT) with high-volume fluid therapy (HVFT). No significant difference in early glomerular filtration rate (GFR) was demonstrated when comparing IGDT and HVFT. However, the IGDT group had better preserved glycocalyx and lower levels of inflammatory markers [106].

The large volume of IV fluids transfused during KTx can be harmful [107,108]. According to a study, kidney recipients who did not require fluid therapy of more than 3000 mL and who had a mean arterial pressure greater than 80 mmHg at the time of transplant reperfusion were less likely to develop DGF [107]. Excessive fluid therapy should also be avoided during the postoperative period to prevent iatrogenic fluid overload, which leads to further graft damage [97]. Large volumes lead to other complications, such as pulmonary oedema, infections, myocardial ischaemia, ileus, and increased mortality [103].

Organ function depends more on blood flow than on blood pressure. There is no optimal noninvasive method of monitoring that can predict fluid responsiveness in patients undergoing KTx. Central venous pressure monitoring is known to be a poor indicator of volume status [103,108].

A meta-analysis of randomised controlled trials indicated approaches that include measurement of cardiac output (CO) and calculation of oxygen delivery as guides for fluid replacement; these methods are associated with decreased mortality and postoperative complications [109].

Noninvasive measurement of CO by oesophageal Doppler (OD) in anaesthetised patients has proven benefits in major abdominal surgery [110], and is recommended by the UK National Institute for Health and Care Excellence (NICE) guidelines for patients undergoing major or high-risk surgery. One study evaluated the use of OD for goal-directed therapy for KTx recipients. The use of OD did not improve immediate graft function; however, the number of postoperative complications caused by fluid overload was limited [103]. Another study reported that OD did not alter the volume of fluid administered or the rate of complications in this group of patients [111].

Transoesophageal echocardiography (TOE) can be a useful diagnostic tool to assess right and left heart function, possible outflow tract obstruction, and the presence of pericardial effusion. It also can be used to obtain CO Doppler measurements. TOE has limitations: it does not allow continuous monitoring, is expensive and time-consuming, and measurements are operator dependent [103].

The use of a pulmonary artery catheter is no longer recommended due to the lack of clinical benefits in high-risk surgical patients compared to standard care, as well as the risk of complications [112].

Noninvasive monitoring of cardiac output and dynamic variation of arterial waveform-derived parameters, such as stroke volume variation (SVV), systolic pressure variation, and pulse pressure variation, may be helpful to provide acceptable trending capabilities to track changes in cardiac output and stroke volume (SV). The patient is considered to be responsive to fluid when SV increases by at least 10–15% in response to fluid expansion. Dynamic analysis of flow parameters is considered the most accurate method for predicting fluid responsiveness [103].

The plethysmography variability index (PVI) is a non-invasive method of circulation monitoring. PVI is an unreliable predictor of fluid responsiveness compared to OD during kidney transplantation [113]. The consensus statement of the American Society of Anaesthesiologists (ASA) Transplant Anaesthesia Committee is that the use of SVV, OD, and PVI is promising but limited in renal transplant surgery [108].

#### 3.3.1. Crystalloids

The ideal crystalloid solution for use in the perioperative period in KTx recipients remains unclear, and current data show high heterogenicity in current practice. In many centers, normal saline is considered the standard crystalloid [108,114]. Administration of balanced electrolyte solutions (e.g., Hartmann’s solution or Plasma-Lyte), which contain bicarbonate precursors and a lower concentration of chloride compared to normal saline, are associated with lower rates of hyperchloremic metabolic acidosis in KTx recipients [115,116]. The administration of hyperchloremic normal saline causes vasoconstriction that reduces the glomerular filtration rate in the graft after reperfusion [117]. Hyperchloraemia was found to have a negative effect on morbidity and mortality in patients after non-cardiac surgical procedures [118]. Patients who received solutions with lower chloride content required significantly lower levels of catecholamines compared to the sodium chloride group. The use of balanced acetate-buffered crystalloids with potassium is safe; the risk of hyperkalaemia increases but by no more than 17%, which does not increase the need for dialysis. Low pH caused by sodium chloride itself may increase serum potassium concentration by displacing it from the cell [117]. However, the Cochrane review concluded that it remains uncertain whether solutions with less chloride, compared to normal saline, lead to better graft outcomes when used in KTx recipients [115].

Balanced crystalloids containing potassium appear to be safe and have a better controlled acid-base balance [119]. ASA strongly recommends balanced crystalloid use because it is associated with a better metabolic profile and equal or lower serum potassium levels, compared to 0.9% sodium chloride, in KTx recipients [108].

#### 3.3.2. Colloids

The most commonly used synthetic colloid solutions are hydroxyethyl starch (HES) and gelatin. HES appears to be an effective volume expander, but only in situations when the glycocalyx is intact [120]. A randomised study compared 6% starch 130/0.4 and 4% gelatin, and their effect on kidney function in liver transplant recipients. Gelatin was associated with more inflammatory reactions, reduced splanchnic oxygen delivery, reduced GFR, and a greater increase in creatinine [120]. It has recently been suggested that starches cause AKI with greater frequency, and even mortality in critical care patients. A meta-analysis that included patients undergoing cardiac surgery, liver transplantation, and other procedures showed no evidence that third-generation waxy maize-derived 130/0.4 HES causes renal impairment in surgical patients [121]. However, the safety of the use of HES in KTx recipients is not well studied. ASA does not recommend the use of HES due to a lack of robust evidence [108].

#### 3.3.3. Albumin

The use of albumin in KTx recipients has not been associated with any advantage compared to crystalloids [108].

We suggest avoiding fluid overload. Cardiac output monitoring should be used to assess fluid responsiveness. Balanced crystalloids should be used.

Key articles: [103,107,108].

### 3.4. Perioperative Glycaemic Control

KTx is often complicated by poorer glycaemic control caused by steroids or newly diagnosed PTDM [122,123]. All patients with DM and 87% of those without DM experience post-transplant hyperglycaemia. All patients with DM, and 66% of patients without DM before transplantation, are treated with insulin after surgery [124]. Risk factors for developing PTDM are well described [124,125].

Several studies have confirmed the relationship between DM and complications in the general population. Surgical site infections were found to be reduced in patients with Hb1c < 7% [126].

Hyperglycaemia requires active monitoring and treatment with IV or subcutaneous insulin [122]. A study comparing two glycaemic control strategies, intensive and standard, in KTx recipients, found no difference in the rate of DGF. Furthermore, patients with intensive glycemic control (blood glucose target 3.9–6.1 mmol/L (70–110 mg/dL)) were found to have an increased risk of long-term graft rejection. Tight control of blood glucose is not recommended in KTx recipients [127].

There are a few groups of drugs that control hyperglycaemia, but no strong evidence was found regarding the efficacy and safety of hyperglycemic treatment after transplantation [128]. The new guidelines recommend the use of metformin in KTx recipients with type 2 Diabetes mellitus. There is not much evidence for the use of sodium-glucose cotransporter-2 inhibitors (SGLT2i) [125,128,129].

KDIGO recommends screening non-DM KTx recipients with fasting plasma glucose, oral glucose tolerance testing, and/or HbA1c at least weekly for four weeks every three months for one year, and annually thereafter. If PTDM develops, the immunosuppressive drug regimen requires modification, balancing the risk of acute rejection against the risk of DM. HbA1c should be targeted from 6.5% to 8.0%, with higher targets for patients with multiple comorbidities and a risk of hypoglycaemia. In the perioperative period, blood glucose should be maintained between 7.8–10 mmol/L (140–180 mg/dL). Patients with PTDM after discharge from hospital should be advised to self-test their capillary blood glucose with a target of 4.4–7.2 mmol/L (80–130 mg/dL), and with a maximum level of 10 mmol/L (<180 mg/dL). Patients with PTDM should receive aspirin for the primary prevention of cardiovascular disease [125,129]. Changing modifiable risk factors and lifestyles is recommended [125].

We suggest maintaining glycaemia within the recommended range of 7.8–10 mmol/L (140–189 mg/dL) in the perioperative period.

Key articles: [125,129].

### 3.5. Temperature Management

In surgical patients, hypothermia is associated with perioperative complications including surgical site infection, coagulopathy, increased transfusion requirements, pain, impaired drug metabolism, adverse cardiac events, and longer LoS [130,131]. There are no specific data on temperature management in KTx recipients, and therefore general rules for the management of surgical patients should be applied.

According to the NICE guidelines, a patient’s temperature should be 36.0 °C or higher before being transferred from the ward to the operating theatre. If the temperature is lower, active warming should be commenced with a target temperature of at least 36.5 °C. Induction of anaesthesia should not be started with a body temperature below 36 °C. The temperature should be recorded prior to the induction of anaesthesia and then monitored every 30 min. The ambient temperature in the operating room should be adjusted to at least 21 °C when the patient is exposed, and can be lowered later when active warming is established. IV fluids should be warmed to 37 °C [132]. Short-term pre-warming before urological procedures decreased the occurrence of hypothermia, which correlated with a lower transfusion rate and a lower prevalence of surgical site infection [131].

In recovery, the patient’s temperature should be measured on admission and every 15 min thereafter. The core temperature should be kept above 36 °C, and active warming used if necessary. On the ward, the temperature should be checked every 4 h and kept above 36 °C. The expected normal temperature range is between 36.5 °C and 37.5 °C [132].

We suggest that the patient’s core temperature should be maintained at 36.5 °C at least intraoperatively. Active warming should be commenced to maintain normothermia. The temperature should be monitored every 30 min intraoperatively, and every 15 min in recovery.

Key articles: [132].

### 3.6. Prevention of Postoperative Delirium

KTx recipients, who are often vulnerable and frail, can experience POD after being exposed to surgery. POD is an acute decline in cognitive function that manifests as inattention, impaired consciousness, disorientation, memory impairment, hallucinations, delusions, or psychomotor disorders. POD may occur in recipients of all ages, but it is more common in frail people and those older than 75 years [63,133,134,135]. POD can occur in up to 50% of older surgical patients and is associated with poor outcomes, prolonged LoS, graft loss, and mortality. The incidence of POD can be reduced by 40% with the proper intervention [133]. Routine premedication with benzodiazepines, anticholinergic drugs, and long fluid fasting time should be avoided. The European Society of Anaesthesiology recommends implementing non-pharmacological prevention measures, such as cognitive orientation, sensory enhancement with visual/hearing aids, noise reduction and good sleep hygiene, avoiding unnecessary internal catheters, reviews of medication, early mobilisation, and good nutrition [63].

The need for anaesthesia in the elderly surgical population (those more than 60 years of age) is increasing. Excessive depth of anaesthesia should be avoided [44,136]. There was a 21% decrease in propofol administration and a 30% decrease in volatile anaesthetic administration when the bispectral index was targeted between 40 and 60 during surgery [136]. BIS-optimised anaesthesia reduced the risk of POD in patients older than 60 years undergoing non-cardiac and non-neurosurgical procedures [136,137]. The use of anaesthetic for non-anaesthetic purposes, for example, treating HTN by increasing the dose of anaesthetic, should be avoided [44].

Early diagnosis of POD plays an important role in effective treatment [63]. The most widely used screening tool is the Confusion Assessment Method for Intensive Care Unit (CAM-ICU) [63].

The use of dexmedetomidine in older patients for non-cardiac surgery decreased the incidence of POD from 23% to 9%. The optimal dose and timing of drug administration require further investigation [138]. Dexmedetomidine is also used to treat POD; randomised trials confirmed faster resolution of POD and fewer days of ventilatory support after use for patients after non-cardiac surgery [137]. There are no data on patients who underwent solid abdominal organ transplantation.

We suggest that the risk of delirium should be minimised. Cerebral monitoring should be used for elderly patients during anaesthesia. We recommend postoperative screening for POD and early management.

Key articles: [63,136,137].

## 4. Postoperative Care

### 4.1. Bed Rest and Early Mobilization

There are limited numbers of studies that present data on the benefits of early mobilisation after surgery. A prolonged lack of physical activity leads to skeletal muscle atrophy, joint contractures, insulin resistance, microvascular dysfunction, systemic inflammation, atelectasis, and pressure ulcers [139]. Intravascular volume decreases during bed rest, which can cause a higher haematocrit and an increased risk of thromboembolism. Elderly patients are the most affected group [3]. Early mobilisation has many advantages. It improves functional status and decreases the duration of hospital stay in elderly surgical patients [140]. After emergency abdominal surgery, patients had a lower risk of readmission to the hospital when they were mobilised early [141].

We suggest early mobilisation after surgery.

Key articles: [139,140].

### 4.2. Nutrition after Surgery

After KTx, some patients may be at acute risk of malnutrition caused by surgical trauma, steroid use, and preoperative starvation; in this situation, their recovery will be poor [45]. Recommendations on post-surgical period requirements for patients after KTx have been published [43,45]. Early oral intake is strongly recommended, and should be offered within 24 h in KTx recipients [43]. Early feeding was well tolerated in 80–90% of patients after bowel resection, and associated with earlier hospital discharge, decreased risk of infection, and improved postoperative hyperglycaemic control [142]. If oral intake cannot meet energy and nutrient requirements, enteral and parenteral feeding should be implemented and malnutrition evaluated [43].

We suggest an early return to oral diet. Enteral and parenteral nutrition should be used when recommended.

Key articles: [43].

### 4.3. Perioperative Pain Control

The ERAS guidelines recommend the use of multimodal analgesia not only to improve postoperative pain control, but also to facilitate early oral intake, mobilization, and accelerated surgical recovery. This approach is based on a combination of opioids, non-opioid analgesics, and regional anaesthesia techniques [143]. The anaesthetist plays an important role in reducing perioperative stress.

Opioids are widely used to control postoperative pain. The pharmacokinetics of short-acting opioids such as fentanyl, remifentanil and alfentanil are unchanged in patients with renal impairment. Morphine and codeine can accumulate active metabolites. Patient-controlled analgesia (PCA) with fentanyl, with its rapid onset, appears to be an effective option, but opioid-based analgesia can cause several side effects. A high level of opioid use after transplantation is associated with an increased risk of graft failure and death. Patients with renal insufficiency who used prescription opioids pre-transplant were more likely to use them post-transplant [144,145,146]. The number of opioid-tolerant surgical patients after KTx is increasing, and it is important to optimise opioid use [146].

Non-opioid analgesics can be combined with opioids and provide better pain control with fewer adverse reactions. Non-steroidal anti-inflammatory drugs (NSAIDs) are commonly used in many types of surgery, but their potential nephrotoxic effect limits their clinical application [147,148].

Paracetamol is safe to use in patients with ESRF and may be used without dose adjustment, but long-term use can cause analgesic nephropathy [149].

Nefopam is a non-opioid analgesic. It does not impair the function of platelets and only 5% is excreted by the kidneys. Nefopam reduces fentanyl consumption and reduces the incidence of drowsiness in patients receiving KTx [150]. The efficacy of nefopam was confirmed in a meta-analysis. In combination with paracetamol, nefopam has a significant morphine-sparing effect in adults after major surgery [151].

Gabapentoids have proven analgesic and opioid-sparing effects in patients with good kidney function. However, this group of drugs is not recommended for use in patients with kidney failure. In combination with opioids, gabapentoids cause serious adverse effects. In dialysis patients, the concomitant use of gabapentoids and opioids is associated with increased mortality and hospitalization [152].

Recent meta-analyses have cast doubt on lidocaine as a systemic analgesic in the perioperative period [153].

Ketamine is used for neuropathic, chronic, and post-surgical pain. It has a 40% opioid-sparing effect on postoperative analgesia [145]. No dose adjustment is necessary in CKD patients [154].

Some analgesics, such as lidocaine and capsaicin, can be applied as patches, and are safe and effective in the treatment of acute and chronic pain when used in a limited area. This approach provides localized analgesia in patients with kidney disease [149].

Magnesium has an analgesic effect induced by blocking NMDA receptors. This action prevents central sensitization caused by peripheral tissue injury. Administration of magnesium during surgery significantly improves postoperative pain control and reduces the dose of other analgesics. Magnesium reduces chronic, neuropathic (including diabetic neuropathy), and opioid-resistant pain [155,156,157].

Regional analgesia techniques significantly improve postoperative analgesia, reduce postoperative opioid and antiemetic requirements, accelerate recovery of bowel function, and facilitate rehabilitation.

Epidural infusion is a well-established technique for major surgeries. However, it can be technically challenging, causing hypotension, bradycardia, and impaired movement of the lower extremities. ESRF patients may have coagulation abnormalities due to uraemia or circulating heparin administered during HD, so vigilance should be maintained. The usage of traditional neuraxial techniques has declined among clinicians.

Continuous surgical site analgesia (CSSA) is an alternative method. A randomised trial was designed to compare CSSA, epidural analgesia, and morphine PCA in patients after open nephrectomy. Epidural analgesia and CSSA decreased postoperative pain, decreased the area of hyperalgesia, and accelerated patient rehabilitation. CSSA significantly reduced residual pain one month after surgery compared to two other groups, and optimised the patients’ physical and mental quality of life three months after surgery [158].

Transversus abdominis plane block (TAP) is one of the more recently described fascial plane blocks that can provide effective postoperative pain relief following abdominal surgeries [159]. This regional technique blocks somatic pain but has little effect on visceral pain [160]. The goal of the block is to place a large volume of local anaesthetic (LA) between the internal oblique and transversus abdominis, where the nerves from T7 to L1 lie. It does not cause typical epidural side effects such as hypotension, bradycardia, and motor block. Single injection TAP blockade significantly decreases the amount of opioids used during the first 24 h after KTx [161]. Continuous TAP infusion reduces morphine requirements by almost 50% after pancreas transplantation [160].

Quadratus lumborum block (QLB) is an alternative way to control postoperative pain. The local anaesthetic blocks the ventral branches of the spinal nerves that pass over the anterior aspect of the quadratus lumborum. QLB has the potential to cover the T7–L1 spinal nerves [162,163]. The efficacy of QLB was compared with the epidural technique. Patients with QLB after laparoscopic nephrectomy consumed a similar amount of morphine in the first 24 h. The QLB group had a higher mean arterial pressure, a similar pain intensity, a similar degree of PONV, a similar degree of sensory and motor blockade, and a shorter duration of urinary catheter usage [164].

Erector spinae block is a promising novel paraspinal technique that is gaining popularity among anaesthetists as it is easily performed and has a low complication rate [165]. LA is injected under ultrasound on the surface of erector spinae muscles. It has an advantage over other interfacial blocks (such as TAP) because the LA spreads to the paravertebral and epidural space. This would block not only spinal nerve roots but also the communicating branches that carry sympathetic fibres, thus relieving visceral and somatic pain [166]. Additional advantages over other regional techniques (QLB or TAP) include the wide analgesic coverage (from T2 to L3). ESP has been described as an effective method of pain management after KTx. After a single injection, the block lasts 24–26 h [167].

Liposomal and non-liposomal bupivacaine have different durations of action. In the first 24 h after surgery, both agents had a similar analgesic effect in patients after donor nephrectomy. Liposomal bupivacaine is superior, lowers maximal pain scores and lowers opioid use up to 72 h, which contributes to a shorter length of stay and lower incidence of nausea and vomiting [168]. A single administration of liposomal bupivacaine for TAP block provides better analgesia compared to continuous TAP infusion of ropivacaine in pancreas transplant recipients [160].

In ERAS protocols for kidney transplantation, catheters providing analgesia were removed before day 2, where day 0 is the day of the surgery [3,5].

A single dose of intrathecal morphine (ITM) combined with PCA has advantages for perioperative pain control. Patients who received 300 mcg of ITM before induction of anaesthesia for open nephrectomy required fewer IV opioids in the postoperative period. Pruritus was a common side effect, with an incidence of 30–100% [169]. A similar reduction in IV opioids was observed in KTx recipients. Delayed respiratory depression is one of the side effects of ITM administration and requires vigilance [170].

Analgesia plans should be created in the preoperative period where comorbidities are identified, options of pain treatment planned and explained, and patients’ preferences and expectations are managed. In some conditions, pain management is more challenging, namely in patients who are younger, female, or have a history of smoking, depression, anxiety, sleep disorders, negative affectivity, preoperative pain, or use of preoperative analgesia. Preoperative assessment allows for the modification of pain strategies in challenging patients, such as patients with chronic pain or those who are taking high doses of opioids preoperatively. Postoperative pain should be assessed using self-reporting subjective scales. Moderate or severe pain must be managed urgently, using an adjusted dose of opioids to allow for functional recovery, mobilising, and coughing [171,172]. An oral route of analgesia should be offered as soon as the patient can eat and drink [5,172].

We suggest opioid-sparing analgesia; multimodal analgesia as a combination of opioids, non-opioid analgesics, and regional anaesthesia techniques; self-reporting scales for pain assessment and urgent management of moderate and severe pain; and oral analgesia as soon as oral intake is possible.

Key articles: [147,149,171].

## 5. Summary and Conclusions

ERAS protocols minimise the rate of complications, reduce LoS, and improve patient satisfaction. This holistic approach can contribute to a reduction in the backlog generated by the pandemic, allow the provision of care to a broader group of patients, and reduce the rate of cancellations. A shorter period of postoperative recovery translates to reduced costs and green anaesthesia. In this review, we suggest a multidisciplinary, holistic, and evidence-based approach to the perioperative care of KTx recipients. A detailed summary of the recommendations is provided in Table 1.

## Figures and Tables

**Table 1:** ERAS recommendations.

ERAS Intervention	Recommendation	Key Articles
Preoperative medical optimisation	Repeat cardiac evaluation, especially in patients with unstable coronary syndromes, decompensated heart failure, significant valvular disease, and arrhythmias. Echocardiography is recommended for patients with ventricular impairment or valvular disease, and patient at risk of pulmonary hypertension. Recipients with PSP greater than 45 mmHg should be assessed by a cardiologist. Pulmonary function testing should be performed in symptomatic patients.	[15,16,17]
Patient education	Patients should quit smoking at least four weeks before surgery, and smokers should be offered nicotine replacement therapy. We recommend cessation of risky drinking for four to eight weeks before surgery. Preoperative frailty screening should be used for risk assessment.	[14,16,29]
Pre-habilitation	KTx recipients should be offered exercise therapy two to three times a week, lasting more than 30 min.	[39,40]
Improving nutritional status	Kidney transplant recipients should be evaluated, and malnourished patients should be referred to a dietician. Diet and exercise advice should be offered to all obese KTx recipients.	[16,45,46,47]
Carbohydrate drink before surgery	A drink containing at least 45 g of carbohydrates should be offered to all patients, except those with diabetes mellitus and anticipated delayed gastric emptying.	[43]
Anaemia correction	Avoid blood transfusion in KTx recipients. Assess response to iron treatment in anaemic patients. Consider ESA treatment for anaemic KTx recipients with Hb levels 90–100 g/L; balance benefits of reducing blood transfusion and risk of side effects. Do not start ESA in patients with iron deficiency.	[53,54,57]
Anxiolysis	Use anxiolytic for anxious patients before anaesthesia. Avoid routine use of sedative agents.	[64,70,74]
Anaesthetic protocol	Administer PPIs or H2 inhibitors for patients with gastroparesis. Avoid anaesthetic agents that accumulate in ESRF. Avoid hypotension during surgery, and aim for MAP targets above 80 mmHg. Consider noradrenaline as vasoconstrictor. Transfuse 250 mL 20% mannitol before reperfusion. Oliguric patients with post-transplant metabolic acidosis should be offered dialysis.	[79,80,81]
Prevention of PONV	Risk stratification and PONV prophylaxis for all patients.	[98,99,100]
Perioperative fluid management	Avoid fluid overload. Use cardiac output monitoring to assess fluid responsiveness. Use balanced crystalloids.	[103,107,108]
Perioperative glycaemic control	Maintain glycaemia within the recommended range 7.8–10 mmol/L (140–180 mg/dL) in the perioperative period.	[125,129]
Temperature management	The patient’s core temperature should be maintained at least 36.5 °C intraoperatively. Active warming should be commenced to maintain normothermia. Temperature should be monitored every 30 min intraoperatively and every 15 min in recovery.	[132]
Prevention of delirium	Minimise the risk of delirium. Cerebral monitoring should be performed in elderly patients during anaesthesia. We recommend postoperative screening for POD and early management.	[63,136,137]
Bed rest and early mobilisation	Early mobilisation after surgery.	[139,140]
Nutrition after surgery	An early return to oral diet. Enteral and parenteral nutrition when recommended.	[43]
Perioperative pain control	Opioid sparing analgesia; multimodal analgesia as a combination of opioids, non-opioid analgesics, and regional anaesthesia techniques; self-reporting scales for pain assessment and urgent management of moderate and severe pain; oral analgesia as soon as oral intake is possible.	[147,149,171]

## Data Availability

Statement not applicable as this review did not report new data.
